# Mining genetic and transcriptomic data using machine learning approaches in Parkinson’s disease

**DOI:** 10.1038/s41531-020-00127-w

**Published:** 2020-09-09

**Authors:** Chang Su, Jie Tong, Fei Wang

**Affiliations:** 1 Department of Population Health Sciences, Weill Cornell Medical College, Cornell University, New York, NY USA; 2 Department of Mechanical and Aerospace Engineering, New York University, New York, NY USA

**Keywords:** Parkinson's disease, Translational research

## Abstract

High-throughput techniques have generated abundant genetic and transcriptomic data of Parkinson’s disease (PD) patients but data analysis approaches such as traditional statistical methods have not provided much in the way of insightful integrated analysis or interpretation of the data. As an advanced computational approach, machine learning, which enables people to identify complex patterns and insight from data, has consequently been harnessed to analyze and interpret large, highly complex genetic and transcriptomic data toward a better understanding of PD. In particular, machine learning models have been developed to integrate patient genotype data alone or combined with demographic, clinical, neuroimaging, and other information, for PD outcome study. They have also been used to identify biomarkers of PD based on transcriptomic data, e.g., gene expression profiles from microarrays. This study overviews the relevant literature on using machine learning models for genetic and transcriptomic data analysis in PD, points out remaining challenges, and suggests future directions accordingly. Undoubtedly, the use of machine learning is amplifying PD genetic and transcriptomic achievements for accelerating the study of PD. Existing studies have demonstrated the great potential of machine learning in discovering hidden patterns within genetic or transcriptomic information and thus revealing clues underpinning pathology and pathogenesis. Moving forward, by addressing the remaining challenges, machine learning may advance our ability to precisely diagnose, prognose, and treat PD.

## Introduction

Parkinson’s disease (PD) is a progressively debilitating neurodegenerative disease that can lead to severe motor and nonmotor dysfunction^1–3^. Although individuals with PD share core phenotypic features, such as bradykinesia, muscle rigidity, and tremor, there is significant heterogeneity that remains incompletely understood. Over the past two decades, genetics and genomics research has revealed significant heritability of this complex disease^4–8^. Increasing number of genetic risk factors (e.g., genes and mutations) have been demonstrated to be associated to PD^9–14^ or PD phenotypes^15–17^. Yet, there still remain extensive gaps in our understanding of the complete heritability and pathology of PD. Today’s high-throughput techniques such as next-generation sequencing (NGS) and microarray have been continuously producing genetic and transcriptomic data of PD patients. As listed in Tables 1 and 2, a set of PD repositories are providing rich genetic and transcriptomic data of the participants^10,18–30^. This leads to a huge opportunity to investigate the disease. In this context, it’s natural to refer to the recently advanced computational technique, machine learning. Compared to the statistical methods which compute a quantitative measure of confidence to identify the correlations, machine learning has demonstrated the capacity in discovering underlying patterns and insight from rich data and hence has the potential to connect genetics and transcriptomics with clinical outcomes using more complex yet accessible approaches^31^. Our objective is therefore to introduce the reader to the field of machine learning and discuss its applications in genetic and transcriptomic data study in PD. Through the survey of existing studies, this review aims to discuss current achievements and remaining challenges, as well as to suggest possible future directions toward developing better machine learning algorithms with which to identify underlying patterns from genetic and transcriptomic data for advancing PD research.

**Table 1:** Parkinson’s disease repositories with genetic data.

Repository	Participant types	Genetic data screened	Other type of data
HBS (USA, Canada)^18^	PD and HC	Targeted sequencing or Asn370Ser, Glu326Lys, Thr369Met genotyping	Motor and nonmotor assessments, biospecimen data, neuroimaging data
DIGPD (France)^10^	PD and HC	Sanger sequencing	Motor and nonmotor assessments
CamPaIGN (UK)^19^	PD and subjects diagnosed with other causes of parkinsonism/tremor	Sanger sequencing; SNP genotype	Motor and nonmotor assessments
PROPARK (Netherlands)^20^	PD	Targeted sequencing or whole exome sequencing	Motor and nonmotor assessments
LABS-PD (USA, Canada)^21^	PD and HC	Targeted sequencing	Motor and nonmotor assessments, biomarkers, imaging data
PICNICS (UK)^22^	PD	Sanger sequencing	Motor and nonmotor assessments
DATATOP (USA, Canada)^23^	PD	Targeted sequencing	Motor and nonmotor assessments
PDBP^24^	PD and HC	NeuroX genotyping	Motor and nonmotor assessments
Penn-Udall (USA)	PD	Targeted sequencing	Motor and nonmotor assessments
PPMI (USA, Europe)^25^	PD, SWEED, and HC	Whole exome sequence	Motor and nonmotor assessments, CSF biomarkers, neuroimaging data
BioFIND (USA)^26^	PD and HC	Whole genomic sequence of the GBA1 gene	Motor and nonmotor assessments, biospecimen data
IPDGC (worldwide)^30^	PD and HC	NeuroX genotyping	Not specified

*CamPaIGN* Cambridgeshire Parkinson’s incidence from General Practitioner to Neurologist, *DIGPD* Drug Interaction with Genes in Parkinson’s Disease, *EMBL-EMI* The European Bioinformatics Institute, *HBS* Harvard Biomarkers Study, *IPDGC* International Parkinson’s Disease Genomics Consortium, *LABS-PD* Longitudinal and Biomarker Study in Parkinson’s disease, *NCBI* The National Center for Biotechnology Information, *PD* Parkinson’s disease, *PDPB* Parkinson’s Disease Biomarkers Program, *Penn-Udall* Morris K Udall Parkinson’s Disease Research Center of Excellence cohort, *PICNICS* Parkinsonism: Incidence, Cognition and Non–motor heterogeneity in Cambridgeshire, *PPMI* Parkinson’s Progression Marker Initiative, *PreCEPT* Parkinson Research Examination of CEP–1347 Trial, *PROPARK* PROFIling PARKinson’s disease, *SWEDD* scans without evidence of dopaminergic deficit.

**Table 2:** Parkinson’s disease repositories with transcriptomic data.

Repository	Description	URL
GEO^27^	A public functional genomics data repository, provided by NCBI.	https://www.ncbi.nlm.nih.gov/geo/
ArrayExpress^28^	A public database that stores data from high-throughput functional genomics experiments, provided by EMBL-EBI.	https://www.ebi.ac.uk/arrayexpress/
ParkDB^29^	A complete set of reanalyzed, curated and annotated microarray datasets of Parkinson’s disease.	http://www2.cancer.ucl.ac.uk/Parkinson_Db2/

*GEO* gene expression omnibus.

## Machine learning outline

**Fig. 1:**
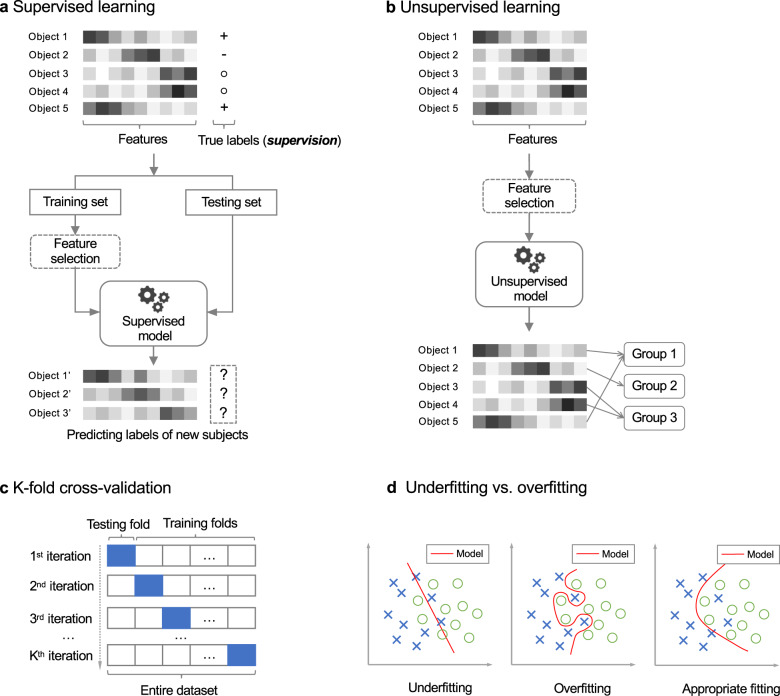
Illustrations of machine learning. **a** An example of supervised learning. A supervised learning model takes input as feature vectors of the subjects and “true” labels of them, a.k.a. supervision information, and contains the following components: feature selection (optional), modeling training on training set, model evaluation on testing set, and model deployment for predicting labels of new data. **b** An example of unsupervised learning. An unsupervised learning model takes input as feature vectors of the subjects only, without any supervision information, and then categorizes the subjects into homogenous groups (a.k.a. clusters). **c** Illustration of the K-fold cross-validation. One by one, each fold is used as testing set, meanwhile one by one, each remaining K-1 folds are used as training set to train model. **d** Illustration of underfitting and overfitting issues. Underfitting occurs when the model doesn’t capture patterns of the data well, while overfitting occurs when the model captures details and noise of training data too well to predict new data correctly.

The term, “machine learning,” is usually used synonymously with “artificial intelligence,” which allows computers to learn from data to uncover patterns and make decisions with minimal human intervention^32^. A central component of machine learning is the supervised learning and unsupervised learning (see Fig. 1a, b).

Figure 1a presents a canonical example of the supervised learning workflow, where we are given a set of data objects to learn from. Each object is represented as an array of measurements commonly called “features”. The array of features is then referred to as a so-called “feature vector.” In a typical supervised learning, each object is associated with a “label,” which can be a class the object belongs to such as diagnosis of PD or not, or a continuous value such as the symptom severity of a patient. The labels are then used as supervision information for training model. In this way, constructing a supervised model typically proceeds with following steps.

After some necessary data preprocessing, a model developer typically splits the data into training and testing sets, then trains the model over the training set by fitting the data using a mathematical function and evaluates the model on the testing set. Though random training-testing splitting has been a common strategy, it may result in sampling bias and fitting the model to a skewed training data. In this context, the cross-validation, especially K-fold cross-validation^33^, has been increasingly engaged. Typically, a K-fold cross-validation divides data into K roughly equal subsets, a.k.a. so-called folds. One by one, each fold is used as testing set, meanwhile one by one, each remaining K-1 folds are used as training set to train model (see Fig. 1c). The cross-validation strategy also helps to evaluate underfitting and overfitting issues, where the former occurs when the model doesn’t capture patterns of the data well and the latter occurs when the model captures details and noise of training data too well to predict new data correctly (see Fig. 1d). Optionally a feature selection procedure can be performed before fitting model to data to enhance the model training. The selected features can be specific ones selected from the original, large set by using statistical testing methods, as well as new, informative ones produced from the original features by using algorithms like Principal Component Analysis. Some models (such as random forest, support vector machine (SVM), and logistic regression) also allow selecting informative features during modeling fitting^34–36^. To evaluate a developed model, usually used performance measurements include accuracy, sensitivity, specificity, and area under the receiver operating characteristic curve (AUC-ROC), which estimates accuracy while comprehensively considering trade-off between true positive rate and false positive rate. Finally, the trained model with the most desirable performance is then deployed to predict unknown labels from new data. In other words, the model “trained” through supervision by labeled data is then used to predict labels of new objects.

For the purpose of accelerating understanding of the molecular biology and pathology of complex diseases, supervised learning has been used in the analysis of genetic and transcriptomic data and has achieved promising results^37,38^. In this context, “features” are genetic factors (e.g., single-nucleotide polymorphisms [SNPs]) or genomic variables (e.g., gene expression levels), and “labels” are disease traits, phenotypes, symptom severities. Some supervised learning models that have been successfully involved in genetics and genomics include logistic regression, Bayesian, decision tree, SVM, k-nearest neighbors (KNN), and neural network models, etc^38^.

In contrast to supervised learning, an unsupervised learning model takes input as feature vectors of the objects only. As shown in Fig. 1b, without any supervision information (i.e., labels), the unsupervised learning model is more straightforward, typically aiming at dividing the input data into homogenous groups (a.k.a. clusters) such that objects within a group have similar patterns somehow and that from different groups are distinct. Such nature makes the unsupervised learning important to the study of the complex diseases like PD, due to that it can, to some extent, overcome the issues with data labeling and heterogeneity of data. A usual application area of unsupervised learning is the data-driven disease subtyping^39^.

Rather than reviewing the taxonomy and algorithms of machine learning models in detail, we focus here on published practices of machine learning in genetic and transcriptomic data analysis in PD. We discuss the practical problems these machine learning models are trying to solve as well as the remaining challenges. For more details of machine learning on genetic and transcriptomic data analysis in general tasks, the readers may consult several previous reviews^37,38,40^.

## Machine learning-based genetic and transcriptomic data analysis in PD

The overarching objectives of machine learning in genetic and transcriptomic data study mainly fall under two general categories: PD outcome study; and PD biomarker identification. Herein, we provide an overview of the existing studies within each category.

### PD outcome study

**Fig. 2:**
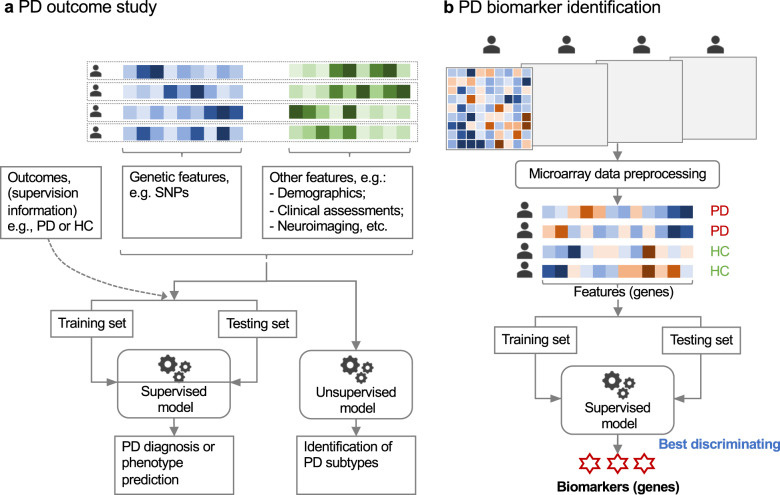
Machine learning in PD genetic and transcriptomic data analysis. **a** Applying machine learning to genetic data (usually combined with other features like demographics, clinical assessments, and neuroimaging features, etc.) for PD outcome study. **b** Applying machine learning to transcriptomic data (e.g., microarray data) for PD biomarker identification.

The use of noninvasive metrics for accurate diagnosis of PD in early stage and prediction of PD phenotypes are promising directions in clinical practice. With the advancement of PD genetics, machine learning models have been more and more engaged to discover heritability from these data (as shown in Table 3). Genome-Wide Association Studies (GWAS) on PD subjects has identified many genetic risk factors such as genetic locus markers, SNPs, variants and alleles^7,9–14,41^. Many studies have directly utilized such risk factors or genetic risk score (GRS) derived from these factors as features to build machine learning models for identification of PD. In addition, since PD has shown to be multifactorial, external information such as demographics, clinical information, and neuroimaging data, are usually combined with genetic factors for integrative analysis of PD patients (as shown in Fig. 2a). For example, Nalls et al.^42^ trained a logistic regression model for classifying PD case patients versus healthy controls (HCs) on the Parkinson’s Progression Markers Initiative (PPMI) population^25^. GRS, together with olfactory function, family history, age, and gender, were selected as predictors by using a greedy feature selection technique known as stepwise regression^43^. Upon the PPMI population, the model achieved an AUC-ROC of 0.92 (95% CI [0.90, 0.95]). The GRS was reported to have a higher predictive contribution than family history, age, and gender. Replication on data from 825 PD patients and 261 controls from five independent cohorts (as shown in Table 3) demonstrated the robustness of the model. Dinov et al.^44^ developed an end-to-end machine learning protocol from data characterization, manipulation, processing, cleaning, and analysis to validation, for PD diagnosis, which flexibly incorporated a series of machine learning models such as AdaBoost^45^, SVM, decision tree, etc. By combining genetic, clinical, demographic, and derived neuroimaging biomarker information from PPMI cohort, their best model achieved an average accuracy over 0.96 of fivefold cross-validation on separating PDs and HCs. Using GRS derived from 1805 variants only, Nalls et al.^12^ achieved an AUC-ROC of 0.69 (95% CI [0.66, 0.73]) in separating PDs and HCs.

**Table 3:** Studies using machine learning for PD outcome analysis.

Study	Task	Discover cohorts	Validation cohorts	Genetic clues/features	Other features	Model
Nalls et al. 2015^42^	PD diagnosis	PPMI: 367 PDs, 165 HCs and 55 SWEDD subjects	PDPB: 453 PDs and 156 HCs; PARS: 15 PDs, 85 HCs and 146 at risk; 23andMe: 20 PDs and 20 HCs; LABS-PD: 239 PDs and 13 SWEED subjects; Penn-Udall: 98 PDs.	GRS from 30 genetic risk factors (28 common risk loci^10^ and 2 rare risk variants, i.e., p.N370S in GBA and p.G2019S in LRRK2)	Olfactory function, self-reported family history of PD, age, sex	Logistic regression
Dinov et al. 2016^44^	PD diagnosis	PPMI: 263 PDs, 40 SWEDD subjects and 127 HCs	None	Not specified.	Clinical data, demographics and derived neuroimaging biomarker data.	A series of typical machine learning methods, such as AdaBoost^45^, SVM, decision tree, etc.
Kraemmer et al. 2016^46^	ICD prediction	PPMI: 276 PDs (86% started DRT, 40% DA, 19% reported incident ICD behavior during follow-up in the study)	None	Genotype of 13 genes: DRD2, DRD3, DAT1, COMT, DDC, GRIN2B, ADRA2C, SERT, TPH2, HTR2A, OPRK1, and OPRM1.	Age, sex, PD treatment (no treatment, DA treatment, other DRT), and duration of follow-up.	Logistic regression
Latourelle et al. 2017^47^	Motor progression prediction	PPMI: 312 PDs and 117 HCs	LABS-PD: 317 PDs	53 a priori selected PD-related SNPs, 17403 SNPs by LD pruning and 10 genetic principal components.	7 CSF protein biomarkers, 8 DaTscan imaging variables and 18 clinical and demographic variables.	Ensemble model based on Bayesian platform
Liu et al. 2017^49^	GCI prediction in PD	HBS: 556 PDs; PDBP: 499 PDs; CamPaIGN: 114 PDs; PICNICS: 129 PDs; PROPAR: 327 PDs; DIGPD: 409 PDs.	DATATOP: 437 PDs; PreCEPT: 332 PDs; PPMI: 396 PDs	GBA mutation status	Age at onset, sex, years of education at baseline, baseline MMSE, MDS-UPDRS II and III scores, Hoehn and Yahr stage, and baseline depression status	Multivariable Cox regression model
Tropea et al. 2018^50^	Cognitive decline prediction	100 PDs	None	APOE, COMT, MAPT variants and GBA mutations	Biomarkers from clinical, biochemical (CSF), and MRI-based imaging modalities	Multivariate linear mixed-effects model
Nalls et al. 2019^12^	PD prediction	IPDGC: 5,851 PDs and 5,866 HCs.	HBS: 527 PDs and 472 HCs	GRS form 1805 variants.	None	Not specified
Fereshtehnejad et al. 2017^51^	PD subtyping	PPMI: 421 PDs	None	GRS from 30 genetic risk factors (28 common risk loci^10^ and 2 rare risk variants, i.e., p.N370S in GBA and p.G2019S in LRRK2)	Demographics, motor manifestations, neuropsychological testing, and other nonmotor manifestations	Unsupervised learning model

*APOE* Apolipoprotein E, *CamPaIGN* Cambridgeshire Parkinson’s incidence from General Practitioner to Neurologist, *COMT* Catechol-O-methyltransferase, *CSF* cerebrospinal fluid, *DA* dopamine agonists, *DATATOP* deprenyl and tocopherol antioxidative therapy of parkinsonism, *DIGPD* Drug Interaction with Genes in Parkinson’s Disease, *DRT* Dopamine replacement therapy, *GBA β*-glucocerebrosidase, *GCI* Global cognitive impairment, *GRS* Genetic risk score, *HBS* Harvard Biomarkers Study, *HC* Healthy control, *ICD* Impulse control disorder, *IPDGC* International Parkinson’s Disease Genomics Consortium, *KNN* K-nearest neighbor classification, *LABS-PD* longitudinal and Biomarker Study in PD, *LD* linkage disequilibrium, *MAPT* microtubule-associated protein-tau, *MDS-UPDRS* Movement Disorders Society-Unified Parkinson’s Disease Rating Scale, *MMSE* Mini Mental State Examination, *PARS* Parkinson’s Associated Risk Study, *PD* Parkinson’s disease, *PDPB* Parkinson’s Disease Biomarkers Program, *PICNICS* Parkinsonism: Incidence, Cognition and Non–motor heterogeneity in Cambridgeshire, *PPMI* Parkinson’s Progression Marker Initiative, *PROPARK* PROFIling PARKinson’s disease, *SWEDD* scans without evidence of dopaminergic deficit.

Furthermore, there are some studies focusing on predicting the phenotypes of PD. For example, in order to predict impulse control disorder (ICD) induced by dopamine replacement therapy, Kraemmer et al.^46^ investigated 13 candidate variants from the DRD2, DRD3, DAT1, COMT, DDC, GRIN2B, ADRA2C, SERT, TPH2, HTR2A, OPRK1, and OPRM1 genes. The results demonstrated that adding these variants as predictors significantly increased ICD predictability (AUC-ROC = 0.76, 95%CI [0.70, 0.83]) compared with the prediction results with clinical variables alone (AUC-ROC = 0.65, 95%CI [0.58, 0.73], *p* = 0.002). Variants of OPRK1, HTR2A, and DDC genes were found to be significant risk factors of ICD. Latourelle et al.^47^ designed an ensemble model to predict annual rate of change in motor signs and symptoms of PD in PPMI and LABS-PD (Longitudinal and Biomarker Study in Parkinson’s disease)^21^ cohorts. Along with demographic, clinical, biomarker, and dopamine transporter SPECT (DaTscan) features, a wide range of genetic data were examined including 53 known PD-related SNPs, 17,403 SNPs identified by linkage disequilibrium pruning from genome, and ten genetic principal components derived from genome^48^. The results showed that these genetic variations were the most predictive to motor progression comparing with other features. In another study, Liu et al.^49^ predicted the progression of global cognitive impairment through longitudinal analysis of the patient data from nine different cohorts. Combined with longitudinal clinical assessments (e.g., MDS-UPDRS Part I-III), mutations in the GBA (*β*-glucocerebrosidase) gene were fed to the proposed machine learning model, which achieved an AUC-ROCs of 0.86 (95% CI [0.82, 0.90]) and 0.85 (95% CI [0.78, 0.91]) in discovery and replication populations, respectively. Tropea et al.^50^ tested for predictors of progression of cognitive decline, and found that the APOE E4 allele was the best predictor.

In addition to PD diagnosis and phenotype prediction where supervised learning is largely performed, data-driven subtyping has a great potential to illuminate underlying pathologies, where an unsupervised learning is more appropriate^39^. One existing study^51^ has incorporated genetic data to identify PD subtypes using machine learning. Combining GRS derived from 28 GWAS loci and two additional risk variants GBA p.N370S and LRRK2 p.G2019S^42^, with demographics and clinical assessments at baseline, an unsupervised learning approach was performed. Subjects with close patterns in terms of genotype and phenotype were gathered into a group, representing a subtype of PD. Three subtypes with distinct PD progression patterns were identified. To date, there hasn’t been much work in the area of applying the unsupervised learning model to genetic and genomic data analysis in PD, probably due to that identification of the groups/clusters is usually subjective and more follow-up efforts are needed for interpreting the identified groups. Yet, it is increasingly attracting attentions as it provides a novel way to discover integrated pattern from genetic and phenotypic information and is promising to personalized medicine.

From these studies we can observe that the genetic information were usually used as predictors in constructing machine learning models for PD diagnosis, phenotype prediction, and subtyping. In many cases, the genetic information have demonstrated to be more indicative than other clinical features. Even so, it is often the combination of genetic and clinical features that can create more robust models overall, compared to the ones based on genetic or clinical features only.

### PD biomarker identification

Identifying biomarkers is critical to the early diagnosis, disease prevention, as well as medication response assessment. These in turn will advance efforts to design and interpret disease-modifying clinical trials that use biomarkers for participant enrollment or as outcome measures^52,53^. A scenario where machine learning models can be applied is discovering the combination of multiple genes whose expression levels in a tissue of interest can discriminate PD patients from HCs, or different phenotypes of PD. Such genes together, constructing a so-called gene signature, may illuminate disease biology and if highly predictive, may provide reliable biomarkers. There have been machine learning models developed for such purpose using the transcriptomic data, e.g., microarray data^38^. As shown in Fig. 2b, machine learning generally transforms the problem into identifying genes as predictors that comprise the model with the best PD vs. HC predictive performance. Each microarray chip can simultaneously measure expressions of thousands of genes in a tissue of interest, e.g., brain or blood. In preparation, a first and necessary step is preprocessing the microarray data, which usually includes one or both of the following operations: removing low-intensity probes or genes; using statistical approaches such as analysis of variance (ANOVA) to originally select differentially expressed genes as candidates of biomarkers. Next, after splitting subjects into training and test sets, the machine learning classifier model is trained over the training set and evaluated for its ability to discriminate PDs from HCs over the test set. Then biomarkers may be determined by selecting predictors (i.e. genes) associated with a top classification performance, e.g., reaching a higher AUC-ROC value. Finally the identified biomarkers are validated by using an independent validation set^54,55^ or by using qPCR^56^ to detect DNA copy numbers and RT-qPCR^57^ to validate RNA expression levels of target genes.

Machine learning has been used to analyze transcriptomic data in PD and demonstrated its capacity to distinguish PDs from HCs^58^. To date, using the machine learning based framework, researchers have identified a number of biomarkers for PD from the transcriptomic data (see Table 4). For instance, Scherzer et al.^54^ investigated 105 individuals by scanning genome-wide expression changes in blood and trained a machine learning model to discriminate PDs from HCs, where 8 genes were identified as candidate biomarkers including VDR, HIP2, CLTB, FPRL2, CA12, CEACAM4, ACRV1, and UTX. Follow-up studies have also successfully identified multi-gene biomarkers from blood samples that are highly indicative of PD^57,59,60^. In addition, by using other neurodegenerative diseases that have overlapping clinical phenotypes with PD (e.g., Alzheimer’s disease [AD] and atypical parkinsonian disorders [APD] or Lewy Body Dementia) as control cohorts, there were studies demonstrated that the identified biomarkers are robust and have great potential for helping reduce misdiagnosis^55,56,61^.

**Table 4:** Studies based on machine learning for PD biomarker identification.

Study	Genetic data	Participants	Model	Validation	Biomarkers
Scherzer et al. 2007^54^	Microarrays from whole blood samples	50 PDs and 55 HCs	Supervised classification model	Validation sample set	8 genes: VDR, HIP2, CLTB, FPRL2, CA12, CEACAM4, ACRV1, and UTX
Molochnikov et al. 2012^55^	Microarrays from blood samples	38 de novo PDs, 24 early stage PDs (within 1st-year medication), 30 advanced PDs, 29 ADs and 64 HCs	Stepwise multivariate logistic regression model	Validation sample set	5 genes: HIP2, ALDH1A1, PSMC4, HSPA8 and EGLN1
Potashkin et al. 2012^56^	Splice variant-specific microarrays	51 PDs, 34 APD (17 MSA and 17 PSP), 39 HCs	KNN	qPCR	13 genes: C5ORF4, WLS, MACF1, PRG3, EFTUD2, PKM2, SLC14A1-S, SLC14A1-L, MPP1, COPZ1, ZNF160, MAP4K1 and ZNF134
Santiago et al. 2013^59^	Gene expression data from blood samples	50 PDs and 46 HCs	Stepwise multivariate LDA	None	7 out of 13 genes in previous study^56^: C5ORF4, COPZ1, MACF1, WLS, PRG3, ZNF160 and EFTUD2
Karlsson et al. 2013^60^	Microarrays from blood samples	79 PDs and 75 HCs	CPLS	None	6 genes: LRPPRC, BCL2, SRSF8, HSPA8, UBE2K, EGLN1
Calligaris et al. 2015^57^	Microarrays from blood samples	52 PDs and 32 HCs	PLS-DA and LDA (a Bayesian classification method)	RT-qPCR	54 genes
Shamir et al. 2017^61^	Microarrays from blood samples	205 PDs, 233 HCs and 48 other neurodegenerative diseases	SVM	None	A gene signature of 87 gene (64 upregulated and 23 downregulated genes between PD and HC)

*AD* Alzheimer’s disease, *APD* Atypical parkinsonian disorders, *CPLS* canonical partial least squares, *HC* Healthy control, *KNN* K-nearest neighbor classification, *LDA* linear discriminant regression (a Bayesian classification method), *MSA* multiple system atrophy, *PD* Parkinson’s disease, *PSP* progressive supra-nuclear palsy, *qPCR* quantitative polymerase chain reaction, *SN* substantia nigra, *SVM* support vector machine.

From these studies we can observe that machine learning approaches has been used to analyze the transcriptomic data in PD and has demonstrated its capacity in advancing development of potential PD biomarkers.

## Discussion: limitations and future directions

We summarize remaining limitations and challenges that the reviewed studies suffered from, and accordingly discuss potential future directions which may lead to promising machine learning approaches to address the issues (see Table 5).

**Table 5:** Summary points of challenges and potential future directions to address them.

Challenges	Potential future directions
Bias of sample size	Integrated multiple cohort modeling.
Handling whole spectrum genetic information	Engaging appropriate feature engineering tools such as genetic principal component analysis^48^, multidimensional scaling^64^, linear discriminant analysis^65^, etc.; Incorporating appropriate deep learning model such as autoencoder^66^.
Multifactorial modeling	Multivariate modeling; Incorporating kernel approaches and probability models.
Cohort diversity	Validation on an external cohort; Training model on data from multiple populations if possible; Engaging transfer learning.
Model interpretation	Using interpretable models such as Bayesian, rule-based (e.g., decision tree and random forest), logistic regression models, etc.; Incorporating or developing model interpretation methods for “black box” models, e.g., deep learning models.
Model evaluation	Evaluation using isolate validation data set; Applying experimental test evaluation; Developing visualization tools for model evaluation.
Interdisciplinary issue	Deep interdisciplinary collaboration; Incorporating domain knowledge in model training.

### Bias of sample size

In many machine learning applications, a common stumbling block to biological and medical domains is that the sample size is insufficient to achieve adequate power. For example, among all microarray data sets used for identification of biomarkers, to our knowledge the largest study in which an SVM (i.e., support vector machine) model was trained using only 205 PD subjects, 233 HCs and 48 subjects with other neurodegenerative diseases^61^. Genotyped subject cohorts with rich clinical data such as PPMI^25^ and BioFIND^26^ are also limited (~470 PD subjects, 80 subjects of SWEDD [i.e., scans without evidence of dopaminergic deficit] and 230 HCs for PPMI, and ~130 PD and 100 HCs for BioFIND). However, in many scenarios where machine learning has achieved clinically useful insights, we need tens or even hundreds of thousands of samples. An undesirable consequence of training on small-size data is that models can easily overfit to the data and it is then hard to generalize to new subjects. Creating a large patient cohort would be ideal but is expensive and time-consuming. Nowadays there are quite a few publicly available cohort repositories from observational studies containing both the genetic and clinical information^10,18,19,21–23^. Therefore, it would be highly valuable to develop machine learning approaches that can integrate multiple such cohort data. There are some existing studies trying to leverage multiple datasets in the learning process^42,47,49^, where the model is still trained on a single data set and the other data sets are mainly for replication purpose. Ideally, data from different repositories should be appropriately combined from which the machine learning model can be learned more robustly. AMP-PD (Accelerating Medicines Partnership: Parkinson’s Disease, https://amp-pd.org) established a knowledge portal which harmonized clinical, genetic, and transcriptomic data of four cohorts, PPMI, BioFIND, PDBD (Parkinson’s Disease Biomarker Program), and HBS (Harvard Biomarkers Study), hence provides the potential of applying a larger-scale machine learning-based study on PD genetic and transcriptomic data.

### Handling whole spectrum genetic information

GWAS has successfully identified hundreds of genetic risk factors associated with traits of PD, however the factors identified so far only capture a small portion of the heritability and even an aggregation of these effects is often not predictive enough for clinical utility. This issue refers to “missing heritability” in which effect sizes of individual factors are too small to pass the stringent significance filters used in many studies^62,63^. In their current stage, machine learning models simply utilized GWAS identified genetic risk factors or GRS derived from them to make up the feature vectors. Existing studies demonstrated that such classifier is accurate enough in a cohort study. However, if we want to train the most accurate possible model that can capture “missing heritability” and can be generalized to new subjects, using only known risk factors as predictors will not suffice. There remains a need to incorporate whole-exome or even whole-genome information. In addition, when we apply analysis with the use of multiple platforms, we usually have to aggregate datasets that are generated using different sequencing technologies, which may incorporate many cleaning and calling issues that make the results unreliable and noisy. In this context, analyzing whole spectrum genetic information also helps to address such issues. Latourelle et al.^47^ has made an attempt to investigated a wide range of genetic information, including known risk SNPs, genome-wide SNPs, and genetic principal components derived from genome^48^. In addition, many optional feature engineering techniques have also been developed to reducing dimensionality of data. The state-of-the-art methods include multidimensional scaling^64^, linear discriminant analysis^65^ and autoencoder^66^, etc. Especially, autoencoder^66^, an important subcategory of deep learning, has shown impressive effectiveness and efficiency in generating low-dimensional representation from extreme high-dimensional data such as genome-wide expression data^67^. These approaches should be examined for their utility in future work.

### Multifactorial modeling

It has been clearly demonstrated that PD is a multifactorial disease, therefore PD prediction or forecast in its early stage needs to comprehensively consider multivariate information. How to aggregate heterogeneous information, such as genetic, genomic, clinical, neuroimaging, social demographic and environmental exposure data, poses a big challenge to conventional computational approaches. The most straightforward way to handle heterogeneous data is to convert each type of data into vector format prior to processing, and orderly concatenate all vectors specific to each subject into a long vector. This has been the most common approach to current PD diagnosis and phenotype prediction. For example, in addition to genetic data, Nalls et al.^42^ incorporated demographics, olfactory function and self-reported family history of PD; Dinov et al.^44^ further used clinical and derived neuroimaging biomarker data; Latourelle et al.^47^ additionally utilized CSF protein biomarkers, etc. By modeling multifactorial aspects of PD, all these methods achieved high performance value (AUC-ROC over 0.80). On the other hand, the kernel approaches^68^ and probability models^69^ are alternative techniques that can fuse heterogeneous data and can be introduced to address this issue in the future.

### Cohort diversity

Like other diseases, the cohort diversity issues may impact the different aspects of PD research (e.g., clinical trial design^70^), where machine learning is expected to play an important role. For example, one potential diversity problem with the current cohorts is ethnicity, as most existing genetic and genomic studies are highly skewed toward the European ancestry^71^. Genetic factors have been found to change their roles in PD risk in different ethnicities (e.g., differences in genetic risk factors have been found between the European and Asian populations^72^). Another is the clinical diversity. For example, PPMI recruits early-stage untreated PD patients^25^; while BioFIND recruits patients who are in moderate to advanced stages^26^. These diversity issues may hinder the generalizability of the developed machine learning models. For example, a model developed on the European cohort may not perform well on the African or Asian cohort, and a model trained from PPMI may not work for BioFIND without any adaptations. In this context, the PD community has taken great steps toward addressing the cohort diversity issues. The International Parkinson Disease Genomics Consortium^71^ and the Global Parkinson’s Genetics Program (GP2, https://parkinsonsroadmap.org/gp2/) have initiated efforts in analyzing data and samples worldwide. AMP-PD is also a good example that provides the platform for facilitating cross-cohort investigation. On the other hand, the model developer and end-user should be mindful of these cohorts’ diversity issues. First, a well-behaved model on a single population is not sufficient, and external validation on independent cohorts is needed. Second, training the model with data from multiple cohorts is always a good choice whenever possible. Third, new machine learning strategies like transfer learning^73^ could be potentially helpful here. Instead of directly duplicating the entire model, transfer learning typically fine-tunes the model parameters trained on one cohort in another cohort, which thus leverages the knowledge from both cohorts.

### Model interpretation

A long-standing concern of machine learning, especially in medicine, is the model interpretation, because that not only the model’s prediction performance but also the clues for making the decision are essential. For example, in biomarker identification, a researcher would expect to see the contribution of expression level of a specific gene in discriminating PD and HCs, indicating why the gene was or was not selected as a biomarker by the model. In this context, the traditional machine learning models, including Bayesian, rule-based models (e.g., decision tree and random forest), logistic regression, SVM, etc., are instinctively capable to estimate feature contributions while training the models. This could be one reason why most of the reviewed studies rely on these approaches. Importantly, some models (e.g., SVM and logistic regression) can be extended to contain the nature of selecting informative features in two ways: (1) plus a regularization term to reduce contribution of a noninformative feature to zero^34,35^; or (2) being embedded in a wrapper such as greedy forward wrappers^36^. This results in the pipeline integrating model training, evaluation, and interpretation in an end-to-end manner.

In addition, deep learning, a new branch of machine learning, has made impressive advances in computer and data science^74^. A deep learning model usually appears to be a “black box” model due to its high complexity. Though preliminary studies have reported a greater computational capacity and flexibility of deep learning in genetics and genomics^38,75^ as well as health care^76^, it’s encountering a larger challenge in model interpretation. There have two potential strategies addressing this issue: to measure changes in model output while involving systematic modification of the input^77^; or to engage third-party tools to determine the feature contributions^78^. Solving the issue, PD genetic and transcriptomic data analysis may largely benefit from the prominent deep learning models.

### Model evaluation

Model evaluation is essential for machine learning model development. Quantitatively, model evaluation tells us how the model performs with measures such as accuracy, sensitivity, specificity, AUC-ROC, etc. Typically, multiple random training-testing splitting or K-fold cross-validation are performed and the average performance along with standard deviation and statistical significance are reported. Yet, evaluation is limited to data in hand and it is hard to keep model performance consistent when encountering new data. To address this, researchers have tried to use isolated data sets to evaluate models trained on discovery sets^42,47,49^. In addition, experimental tests are also useful in model evaluation. For example, qPCR^56^ and RT-qPCR^57^ were used to validate gene expression of identified biomarkers. Such domain experts guided evaluation may enhance model confidence significantly. Alternatively, qualitative evaluation is another way for model evaluation, which often engages tools to demonstrate machine learning findings to enhance stability and interpretability of the produced model, such as visualizations of feature importance and comparisons of characteristics of identified subtypes. It helps to understand machine learning outcomes intuitively.

### Interdisciplinary study

In practice, for effective application of machine learning methods to achieve good performance, good understanding on both machine learning methodology and domain-specific knowledge is necessary. Standing at the crossroads of genetics, transcriptomics, PD, and machine learning fields, it is challenging for researchers to solve this interdisciplinary problem. For neurologists in particular, it is challenging to be aware of the mathematical background of machine learning models to proficiently develop algorithms. For machine learning developers, knowing less about genetics, genomics and PD hinders designing the best model that can appropriately organize genetic and transcriptomic data as well as fully incorporate domain knowledge. In developing the most appropriate model, neurologists need to play a crucial role in the entire development life cycle, from coming up with conception practical and impactful applications, and providing domain knowledge to guide model building, to model evaluation before practice in clinic. To this end, deep collaboration between neurologists and machine learning developers is highly recommended.

Another flexible way to address the interdisciplinary issue is to incorporate prior domain knowledge to guide the model to learn. In this context, previously identified genotype-phenotype correlations can be used as domain knowledge to enhance PD predictive modeling. For instance, individuals with PD due to parkin (PARK2) gene mutations are found to be more prone to levodopa-induced dyskinesias^79,80^, and GBA variants have been associated with a more rapid progression of cognitive dysfunction and motor symptoms of PD patients^16^. Injecting such prior associations into a machine learning model may improve the model robustness in terms of both computability and interpretability. In this context, domain experts play a key role in model development by providing specialized guidance to design rule of model. In addition, gene-gene interactions are also important genomic domain knowledge which may help genetic data modeling and can be downloaded from existing public databases, such as KEGG^81^ and BioGRID^82^. To handle injection of domain knowledge, powerful techniques have been extensively developed, such as kernel^68^ and knowledge embedding^83,84^ approaches.

## Conclusions

Recent years have seen a clear acceleration in our knowledge and ability to apply machine learning models to genetic and transcriptomic data in PD study. Machine learning models that combine genetic data with demographic, clinical and neuroimaging information have achieved significant refinement in PD diagnosis and disease phenotype prediction, as well PD subtype identification. In addition, many potential biomarkers in terms of gene expression levels have been identified through the use of machine learning models. Intrinsic superiority and current achievements of machine learning has demonstrated its promise in genetic and transcriptomic data analysis for advancing PD. However, remaining limitations of current studies are challenging machine learning approaches to make further breakthroughs in thoroughly understanding pathogenesis of the disease. For future research, developing appropriate machine learning models by addressing the issues may lead to great improvements in PD management.
